# Autophagy fine-tuning by angiotensin-(1-9) in cultured rat cardiomyocytes

**DOI:** 10.3389/fcvm.2025.1408325

**Published:** 2025-03-12

**Authors:** Mario Bustamante, Clara Quiroga, Georthan Mancilla, Wileidy Gomez, Anita Tapia, Reinaldo Figueroa, David Mondaca-Ruff, Ingrid Oyarzún, Hugo E. Verdejo, Sergio Lavandero, Pablo Castro

**Affiliations:** ^1^ Advanced Center for Chronic Diseases (ACCDiS), University of Chile & Pontifical Catholic University of Chile, Santiago, Chile; ^2^ Laboratorio de Señalización Cardiovascular, División de Enfermedades Cardiovasculares, Facultad de Medicina, Pontificia Universidad Católica de Chile, Santiago, Chile; ^3^ Physiology and Biophysics Program, Faculty of Medicine, University of Chile, Santiago, Chile; ^4^ Department of Biochemistry and Molecular Biology & Department of Cardiovascular Medicine, Mayo Clinic, Rochester, MN, United States; ^5^ Laboratorio de Transducción de Señales Moleculares, Facultad de Ciencias Químicas y Farmacéuticas & Facultad de Medicina, Universidad de Chile, Santiago, Chile; ^6^ Department of Internal Medicine/Cardiology, University of Texas Southwestern Medical Center, Dallas, TX, United States

**Keywords:** angiotensin-(1-9), autophagy, cardiomyocyte, heart, cardiovascular diseases

## Abstract

**Background:**

The renin-angiotensin system (RAS) plays a pivotal role in regulating blood volume, systemic vascular resistance, and electrolyte balance, serving as a key component of cardiovascular health. Recent findings highlight the role of angiotensin II (Ang II) in inducing autophagy through angiotensin II receptor type 1 (AT1R). Autophagy, a process of self-degradation and turnover of cellular components, is a homeostatic response that eliminates superfluous materials. Abnormal autophagy promotes cardiomyocyte loss and is critical in hypertrophy and heart failure progression. The RAS's non-canonical axis, which includes the angiotensin 1-9 peptide [Ang-(1-9)], has an anti-hypertrophic effect in cardiomyocytes via an unknown mechanism**.** In the present study, we aimed to elucidate the effect of Ang-(1-9) on cardiomyocyte autophagy.

**Methods:**

We **i**solated and cultured neonatal ventricular cardiomyocytes and then **co-**treated them with Ang-(1-9) in the presence of chloroquine (CQ), Ang-II, and chemical inhibitors of different signaling pathways. After treatment, total RNA and protein extracts were obtained to analyze the abundance of different autophagy markers. Likewise, cells were fixed, and autophagy was analyzed through epifluorescence microscopy.

**Results:**

Our findings show that CQ leads to a reduction in autophagy markers, such as microtubule-associated protein 1 light chain 3-II (LC3-II) and total LC3, suggesting Ang-(1-9)'s regulatory role in basal autophagy levels. Furthermore, Ang-(1-9) opposes Ang-II-induced autophagy and induces the phosphorylation of the S234 residue of Beclin-1 (BCN1) via an angiotensin II receptor type 2 (AT2R)/Akt-dependent pathway.

**Conclusions:**

This reduction of Ang-II-induced autophagy by Ang-(1-9) unveils a novel aspect of its action, potentially contributing to its cardioprotective effects.

## Introduction

The renin-angiotensin system (RAS) regulates the cardiovascular response to physiological triggers such as reduced blood pressure or increased cardiac stress. Dysregulation of the RAS can contribute significantly to the pathogenesis of various cardiovascular disorders (1–4) Within the RAS, the angiotensin II (Ang-II)/angiotensin II receptor type 1 (AT1R) axis is critical for regulating blood pressure and influencing the development of cardiac hypertrophy and fibrosis (2, 5). Beyond this well-known pathway, current research sheds light on the non-canonical RAS axis, which features counterregulatory peptides such as angiotensin-(1-9) [Ang-(1-9)], angiotensin-(1-7) [Ang-(1-7)], and the angiotensin II receptor type 2 (AT2R) (2, 6). Particularly noteworthy is Ang-(1-9), a nonapeptide derived from Ang-I via angiotensin-converting enzyme 2 (ACE2) activity, recognized for its cardioprotective effects (7–9). Elevated levels of Ang-(1-9) have been associated with improved outcomes in myocardial infarction and hypertension, notably after the administration of angiotensin-converting enzyme (ACE) inhibitors or AT1R blockers (1, 2, 10–13), highlighting its potential therapeutic value in cardiovascular disease management. The cardioprotective effects of Ang-(1-9) are characterized by the inhibition of cardiac hypertrophy, a reduction in fibrosis, and a decrease in cardiovascular inflammation (12, 14–18). These outcomes are believed to be mediated through mechanisms involving AT2R and cardioprotective kinases such as Akt (14, 15, 19, 20).

Macroautophagy (thereafter autophagy) is a fundamental pathway for maintaining cellular homeostasis, playing an essential role in the degradation and recycling of cellular components such as protein aggregates, lipids, and dysfunctional organelles (21). This process, which involves the sequestration of these materials into a double-membraned vesicle known as the autophagosome for subsequent degradation and repurposing, proceeds through distinct phases: initiation, formation of the autophagosome precursor (highlighted by the crucial conversion of LC3-I to LC3-II protein), expansion and maturation of the autophagosome, and its fusion with the lysosome for substrate breakdown and recycling (21, 22). The importance of autophagy in heart health has become increasingly apparent, given its role in preserving the balance of cellular components. Cardiomyocyte loss through apoptosis or necroptosis significantly affects the progression of heart-related diseases, including heart failure (23). While autophagy supports cardiomyocyte health and overall cardiovascular function by clearing damaged cellular elements (24–27), over-activated autophagy may lead to cell death (28). Conversely, insufficient autophagy diminishes the cell's capacity to manage and eliminate damaged proteins and organelles, underscoring the need for a balanced autophagic activity to ensure cardiovascular health and stability.

The interplay between the RAS and autophagy reveals a multifaceted relationship, the nuances of which remain largely unknown. While certain studies indicate that Ang-I-induced autophagy could mitigate cardiac dysfunction, others highlight a protective aspect through Ang-II's suppression of autophagy (29–34). This complexity introduces an intricate layer to understanding how RAS influences autophagic regulation.

Our research posits that Ang-(1-9) modulates autophagy in cultured rat cardiomyocytes, outlining a potential pathway for Ang-(1-9)'s cardioprotective effects. Our findings show that Ang-(1-9), via AT2R, not only suppresses basal autophagy but also counteracts Ang-II-induced autophagy. This contribution enriches our understanding of Ang-(1-9)'s cardioprotective attributes, shedding light on the still obscure relationship between RAS modulation and autophagic processes in cardiac health.

## Materials and methods

### Reagents

Anti-phospho-mTOR (#5536), mTOR (#2983), phospho-p70S6 K (#9234), LC3B (#2775), phospho-Akt (#4060), Akt (#2920), phosphor-ERK-1/2 (#4370), ERK-1/2 (#9107), and Beclin-1 (#4122) antibodies were acquired from Cell Signaling Technology (Danvers, MA, USA). Anti-phospho-Beclin-1-ser295 (#PA5-35394) and phospho-Beclin-1-ser234 (#PA5-35393) antibodies were purchased from Thermo Fisher Scientific (Waltham, MA, USA). Anti-p62 antibody was purchased from Abnova Co. (Jhouzih St, Taipei, Taiwan). Anti-GAPDH (G9545) antibody and Bafilomycin A1 (Baf-A) were purchased from Sigma-Aldrich Co. (St. Louis, MO, USA). Secondary antibodies were obtained from Calbiochem (Burlington, ON, Canada). Protein Phosphatase Inhibitor Cocktail IV and Protein Protease Inhibitor Cocktail (EDTA-free) were purchased from Abcam (Cambridge, MA, USA). Bovine Serum Albumin (BSA) was purchased from Winkler Ltda. (Santiago, Chile). TRIzol Reagent, Fetal Bovine Serum (FBS), Newborn Calf Serum (NBCS), and M-MLV reverse transcriptase were obtained from Thermo Fisher Scientific (Waltham, MA, USA). Bradford's solution and PVDF membranes were from Bio-Rad Laboratories (Hercules, CA, USA). Westar Supernova substrate was obtained from Cyanagen (Bologna, Italy). SensiFAST SYBR Hi-ROX was purchased from Bioline Meridian Biosciences (London, UK). Dulbecco's Modified Eagle Medium (DMEM), Medium 199 (M199), 5-bromo-2′-deoxyuridine (BrdU), Chloroquine (CQ), PD123319, Akt inhibitor VIII (Akti), and U0126 (U0) reagents were obtained from Sigma-Aldrich (St. Louis, MI, USA). Ang-II and Ang-(1-9) peptides were acquired from GL Biochem (Shanghai, China) Ltda. CytoBuster and other organic and inorganic compounds, acids, and solvents were obtained from Merck (Darmstadt, Germany).

### Cardiomyocyte culture and inhibitor treatments

Neonatal rat ventricular myocytes (NRVMs) were isolated from the hearts of 1- to 3-day-old Sprague Dawley rats, as described previously (35). Cells were plated and cultured for 24 h in DMEM: M199 (4:1) containing 5% FBS, 10% NBCS, 100 μM BrdU, and antibiotics. To evaluate different effects, NRVMs were cultivated for 24 h and treated with Ang-II for 24 h, with Ang-(1-9) for 6 h, CQ for 4 h, and PD, Akti-1/2 (Akti), U0 for 30 min. After treatments, cells were harvested in TRIzol or CytoBuster containing phosphatase and protease inhibitors for harvesting mRNA or protein.

### Western blot analysis

NRVMs were washed with PBS and lysed using CytoBuster, a lysis buffer. Protein concentration was determined using the Bradford method. Then, equal amounts of protein from cells were separated by SDS-PAGE (10% polyacrylamide gels), electrotransferred to PVDF (polyvinylidene difluoride) membranes, and blocked with 5% fat-free milk in Tris-buffered saline (pH 7.6) containing 0.1% (v/v) Tween-20 (TBST). Membranes were sequentially incubated with the following primary antibodies: anti-LC3B (1:1000), anti-phospho-Akt (1:2000), anti-Akt (1:2000), anti-phospho-ERK-1/2 (1:2000), anti-ERK-1/2 (1:2000), anti-GAPDH (1:10000) anti-p62 (1:2000) anti-phospho-Beclin-1-Ser295 (1:250) and phospho-Beclin-1-Ser234 (1:250), and horseradish peroxidase-linked secondary antibodies [1:5000 in 5% (w/v) BSA in TBST]. Luminescence was detected using an ECL solution, visualized and digitized using a C-DiGit Blot Scanner (LI-COR Biosciences, Nebraska, USA), and quantified with Image Studio Lite Software (v.5.2; Li-Cor). Protein content was normalized to GAPDH level; phosphoproteins content was normalized to the corresponding total protein.

### Real-time PCR analysis

Total RNA was extracted from cultured cardiomyocytes using the TRIzol reagent. RNA samples were quantified, and their 260/280 absorbance was measured by NanoDrop (Thermo Fisher Scientific, Waltham, MA, USA). Reverse transcription was performed using 1 µg of RNAand M-MLV reverse transcriptase. RT-qPCR was performed using cDNA amplified, specific primers designed for rats (Table 1) and SensiFAST SYBR Hi-ROX Master Mix in a StepOnePlus Real-Time PCR System. Each sample was run in triplicate. Data for each transcript was normalized to Ywas and Hmbs RNA as an internal control, with the 2^-ΔΔCt value method (36–38).

**Table 1:** Primer sequences for PCR.

Targeted gene	Forward primer (5′–3′)	Reverse primer (5′–3′)
LC3B	CGTCCTGGACAAGACCAAGT	CCATTCACCAGGAGGAAGAA
BCN1	TGTGTGCAGCAGTTCAAAGA	CACTGCCTCCAGTGTCTTCA
ATG5	CAACCGGAAACTCATGGAAT	ACAGGACGGAACAGCTTCTG
ATG12	GCCTCGGAGCAGTTGTTTAT	GGACCAGTTTACCATCACTGC
Gabarapl1	TCGTGGAGAAGGCTCCTAAA	TCTCAGGTGGATCCTCTTCC
Ywas	ACTTGACATTGTGGACATCGGA	GTGGGACAGCATGGATGACA

### Immunofluorescence assays

NRVMs were cultured on gelatin-coated coverslips and fixed with PBS containing 4% paraformaldehyde, incubated for 15 min in ice-cold, permeabilized with Triton X-100 0.1% for 15 min, and blocked with 1% BSA in PBS for 1 h. The cells were incubated overnight with antibodies against LC3B (1:400) and then with the secondary antibodies Alexa Fluor 488-conjugated anti-rabbit-IgG (1:600). As co-staining, to tag and visualize F-actin and nuclei, the cells were incubated with red-phalloidin and Hoechst 33,342 (blue), respectively. Epifluorescence images were taken with an Olympus IX71 Inverted Microscope (Olympus, Hicksville, New York, USA). The background was subtracted from images using the ImageJ software.

### Statistical analysis

Data are presented as mean ± SEM, using Student's *t*-test for pairwise comparisons and one-way or two-way analysis of variance with Dunnet's or Tukey's post-test (respectively) for multiple comparisons. The value of *P* < 0.05 was statistically significant. Statistical analysis of all experiments was performed using GraphPad Prism 8 (GraphPad, La Jolla, CA, USA).

## Results

### Effect of Ang-(1-9) on autophagy

**Figure 1:**
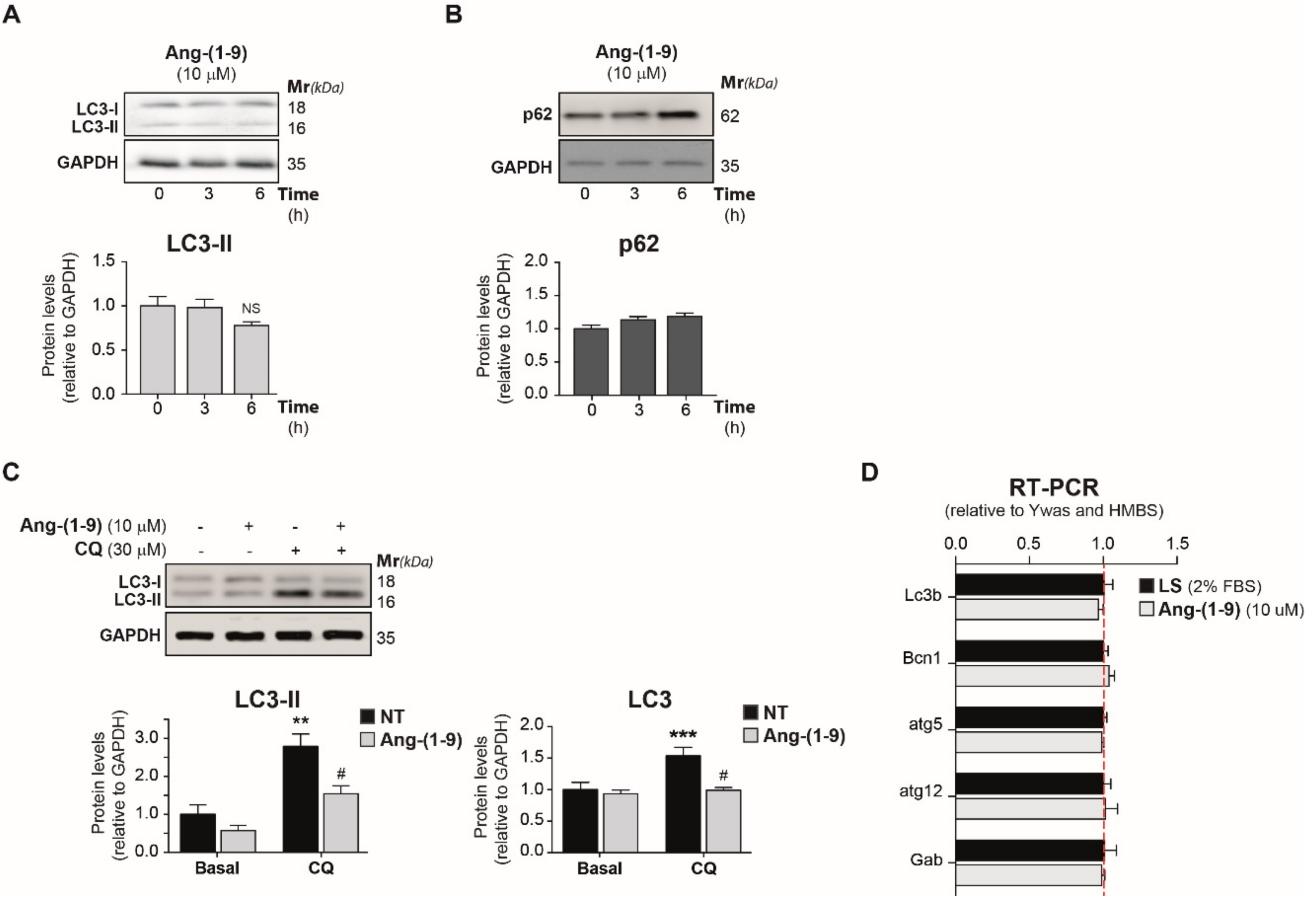
Angiotensin-(1-9) impairs autophagy in neonatal rat ventricular cultured cardiomyocytes. Cardiomyocytes in low serum were treated with 10 μM Ang-(1-9) for 3 and 6 h. Protein levels of LC3-II **(A)** and p62 **(B)** were monitored by Western blot and quantified. The graphs represent the average ± SEM of *n* = 3. No statistical significance was achieved for LC3-II and p62 levels. **(C)** Cardiomyocytes were treated for 24 h with 10 μM Ang-(1-9) in the presence or absence of 30 μM CQ for the last 4 h. Protein levels of LC3-II and total LC3 were monitored by Western blot and quantified. The graphs represent the average ± SEM of *n* = 5. **(D)** Total RNA was purified from cardiomyocytes treated or untreated with 10 μM Ang-(1-9) for 24 h. cDNA was synthesized and Lc3b, Bcn1, Atg5, Atg12 and Gabarapbl1 (Gab) genes were amplified though PCR. Endogenous Ywas and Hmbs genes were used as controls (*n* = 4). **p < 0.01, ***p < 0.001 CQ vs. basal; #*p* < 0.05 Ang-(1-9) vs. NT. NT, non-treated.

Initially, we aimed to evaluate the influence of Ang-(1-9) on baseline autophagy levels. Isolated NRVMs were subjected to treatment with 10 mM Ang-(1-9) for periods of 3 and 6 h, subsequently undergoing lysis for the assessment of autophagy indicators, LC3-II (Figure 1A) and p62 (Figure 1B), via Western blot analysis and LC3-II via indirect immunofluorescence (Supplementary Figure S1A). The treatment with Ang-(1-9) did not result in significant alterations in LC3-II and p62 levels. Since autophagy is a dynamic process, we utilized chloroquine (CQ), which inhibits the fusion of autophagosomes with lysosomes by elevating lysosomal pH. In case autophagy is activated, CQ induces the accumulation of autophagosomes (39), enabling a clear assessment of the autophagic flux. This dual treatment led to a marked reduction in both LC3-II and total LC3 levels (Figure 1C), with a significant 45% decrease in LC3-II protein levels [NT: 1.00 ± 0.26; Ang-(1-9): 0.57 ± 0.14; CQ: 2.79 ± 0.33; Ang-(1-9)/CQ: 1.54 ± 0.21], indicating a potent reduction in the basal autophagic flux by Ang-(1-9). Additionally, to confirm these results, avoiding potential misinterpretations caused by lysosomal neutralization, we use bafilomycin A1 (Baf-A) and we observed a basal reduction in LC3-II levels in conjunction with an increase in p62 (Supplementary Figures S1B,C). This strategy allowed us to corroborate the Ang-(1-9) effect on autophagic flux observed with CQ.

Further analysis through RT-qPCR was conducted to examine if the observed autophagic flux modulation was related to alterations in the expression of essential autophagy-related genes such as Lc3b, Bcn1, Atg5, Atg12, and Gabarapbl1 (Gab). The absence of significant changes in gene expression post Ang-(1-9) treatment points to the peptide's influence on autophagy extending beyond these specific genetic markers (Figure 1D). Thus, our data highlight a suppression of autophagic flux in cardiomyocytes post-Ang-(1-9) administration, observable regardless of gene expression alterations. Consequently, we examined the interaction between Ang-(1-9) and the canonical RAS on autophagy. While the effect of Ang-II on autophagy is extensively documented across various models, such as vascular smooth muscle cells and cardiomyocytes (40, 41), the interplay between Ang-(1-9) and Ang-II in modulating autophagy remains largely unexplored, marking a significant gap in our understanding of their combined impact on cardiovascular health.

### Modulation of Ang-II-induced autophagy by Ang-(1-9)

**Figure 2:**
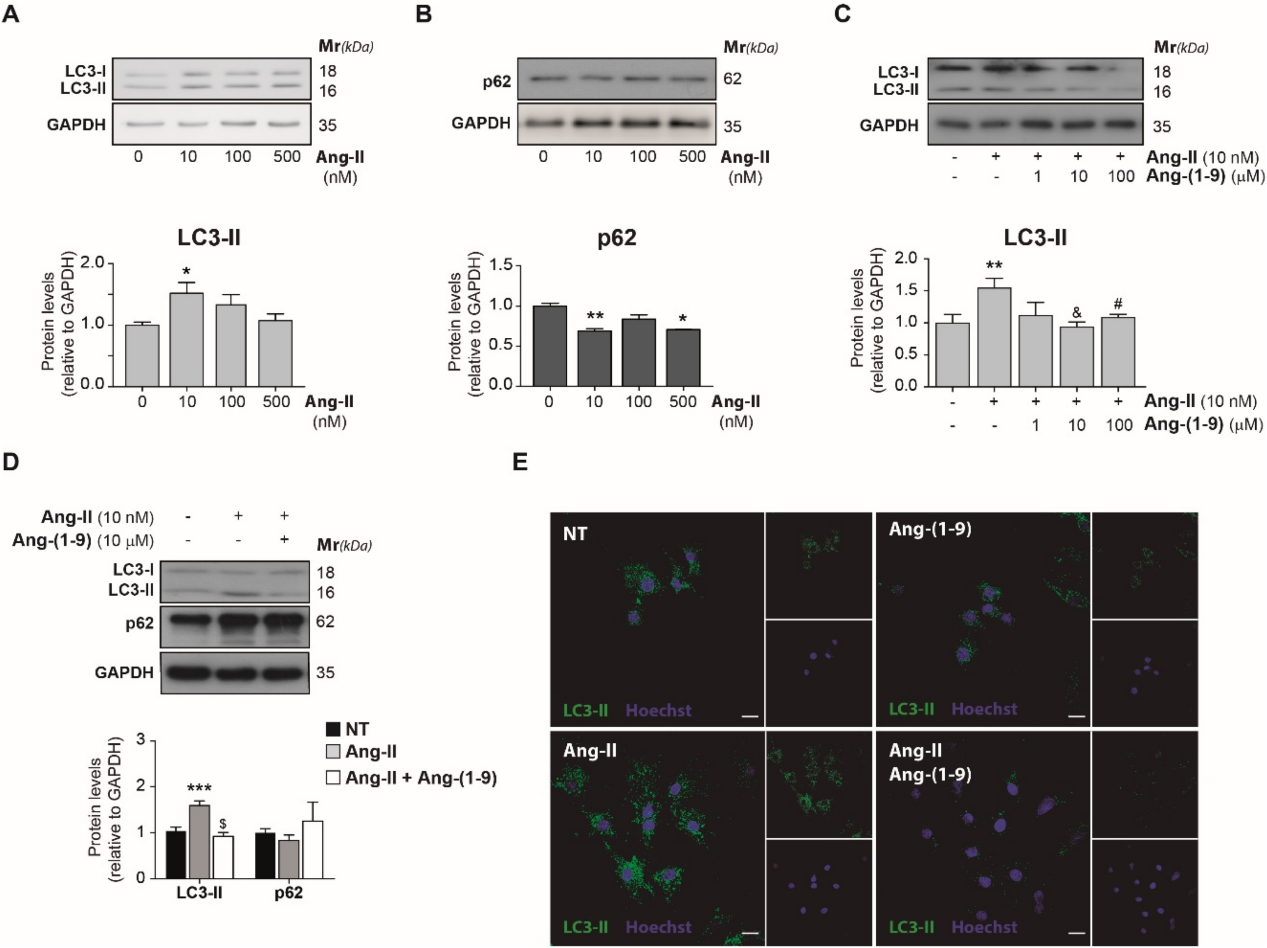
Angiotensin-(1-9) blocks angiotensin II-induced autophagy in neonatal rat ventricular cultured cardiomyocytes. Cardiomyocytes were cultured by 24 h in the presence of different concentrations of Ang-II (10–500 nM) and levels of LC3-II **(A)** and p62 **(B)** were determined through Western-blot and quantified. The graphs represent the average ± SEM. **(C)** Cells were cultured by 24 h with 10 nM Ang II in the presence of different concentrations of Ang-(1-9) (10–100 μM), and LC3-II levels were determined by Western-blot (*n* = 5). **(D)** LC3-II and p62 protein levels at cardiomyocytes cultured by 24 h with 10 nM Ang-II and 10 μM Ang-(1-9) were determined by Western-blot. **(E)** Confocal images of indirect immunofluorescence and detection of autophagosome's LC3 dot pattern (green), and nuclei staining (blue, Hoescht 33342) in cardiomyocytes cultured by 24 h with 10 nM Ang-II and 10 μM Ang-(1-9). Scale bar = 2 µm. Graphs represent the average ± SEM from at least *n* = 3 of protein levels. Data were relativized to GAPDH levels and normalized by NT condition. *p < 0.05 condition vs. 0 h or NT; **p < 0.01 condition vs. 0 h or NT, ***p < 0.001 condition vs. 0 h or NT, #*p* < 0,05 and & *p* < 0.01 Ang-(1-9)/Ang-II vs. Ang-II. One-way ANOVA and Dunnet's post-test.

We investigated the effect of varying Ang-II concentrations (from 10 to 500 nM) on cardiomyocyte autophagy over a 24-h treatment period. Through Western blot analysis, we observed significant changes in the levels of LC3-II (Figure 2A) and p62 (Figure 2B) following treatment with just 10 nM of Ang-II. Specifically, LC3-II protein levels exhibited a roughly 60% increase compared to the basal state, while p62 levels decreased in the presence of Ang-II, signaling the activation of autophagy. To confirm these results, we observed a significant increase in LC3-II levels in the presence of Baf-A (Supplementary Figure S2).

To explore how Ang-(1-9) might modulate Ang-II-induced autophagy, cardiomyocytes were treated with 10 nM Ang-II for 24 h and exposed to varying Ang-(1-9) concentrations, ranging from 10 to 100 μM, during the last 6 h. Treatment with both 10 and 100 μM Ang-(1-9) led to a statistically significant decrease in LC3-II protein levels (Figures 2C,D) when compared to the effects observed with Ang-II alone. An effect that was also observed when autophagosome accumulation was assessed by microscopy (Figure 2E). Particularly in case of p62 protein levels, there was an observed trend towards an increase following Ang-(1-9) treatment, though this change did not reach statistical significance (Figure 2D). These results illustrate the contrasting roles of the canonical and non-canonical RAS on cardiomyocyte physiology, showcasing their complex interplay in modulating autophagic activity.

### Signaling pathways and autophagic modifications induced by Ang-(1-9) in cardiomyocytes

**Figure 3:**
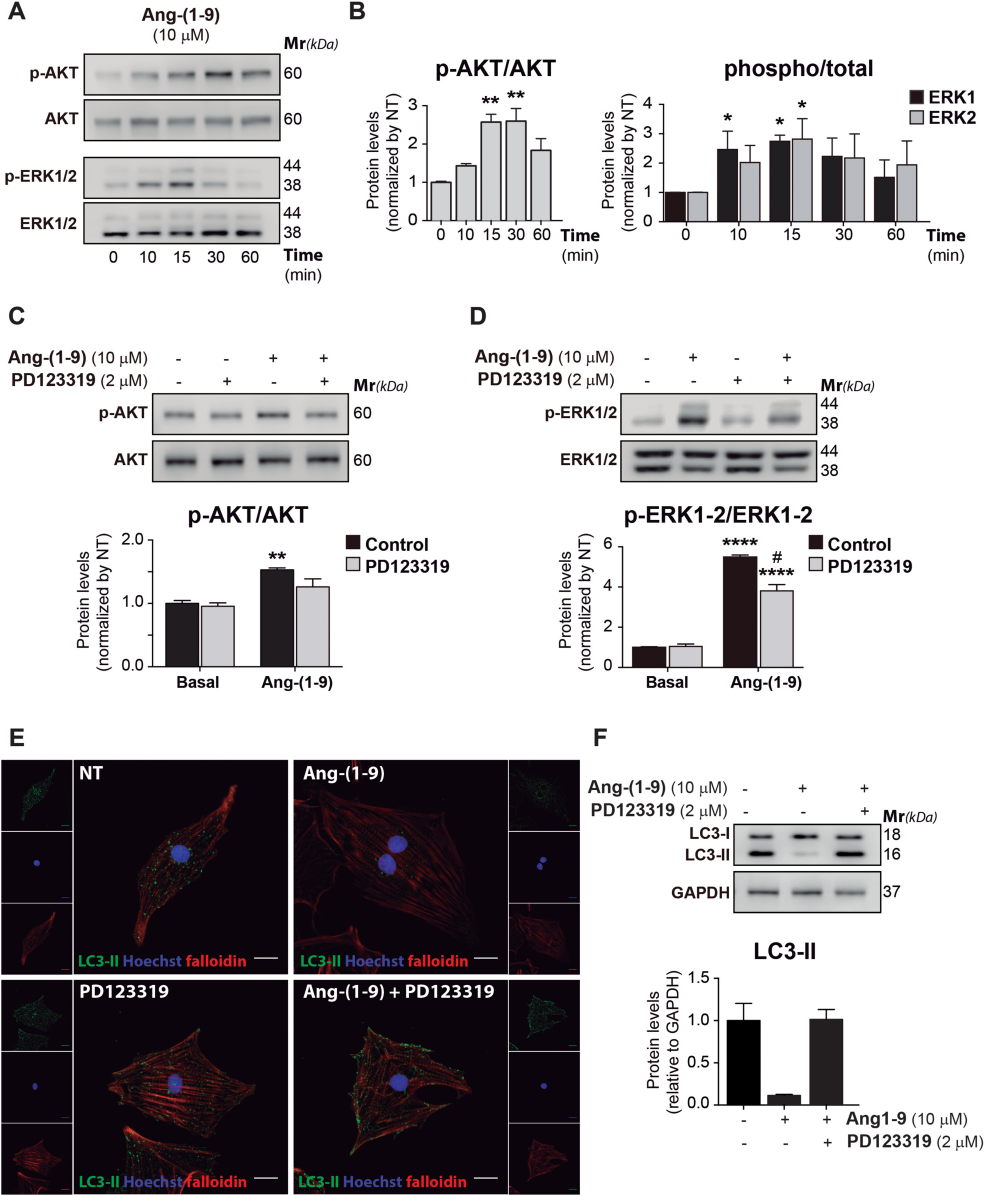
Ang-(1.9) activates the AT2R/Akt/ERK signaling pathway and modifies the cardiomyocyte autophagic tone through AT2R activation. Cardiomyocytes were cultured from 10 to 60 min with 10 μM Ang-(1-9) to determine the phosphorylation of **(A)** AKT and ERK1/2 through Western blot. **(B)** Graphs represent the average ± SEM from *n* = 3 of protein levels. Data were relativized to total AKT and ERK1/2, respectively, and normalized by basal conditions. *p < 0.05 condition vs. 0 h; **p < 0.01 condition vs. 0 h, ***p < 0.001 condition vs. 0 h. One-way ANOVA and Dunnet's post-test. Cells were treated for 30 min with the AT2R antagonist, PD123319 (2 μM) before stimulation with 10 μM Ang-(1-9) for 15 min and **(C)** p-AKT, and **(D)** p-ERK1/2 were determined through Western blot. Graphs represent the average ± SEM from *n* = 3 of protein levels. Data were relativized to total AKT, and total ERK1/2, respectively, and normalized by basal conditions. *p < 0.05 Ang-(1-9) vs. basal; **p < 0.01 Ang-(1-9) vs. basal, ****p < 0.0001 Ang-(1-9) vs. basal, #*p* < 0.05 PD vs. Control. Two-way ANOVA with Tukey's post-test. **(E)** Confocal images of indirect immunofluorescence and detection of autophagosome's LC3 dot pattern (green), actin cytoskeleton (red, phalloidin), and nuclei staining (blue, Hoescht 33342) in cardiomyocytes cultured by 24 h with 10 μM Ang-(1-9) in the presence or absence of 2 μM PD123319 (PD). Scale bar = 2 µm. **(F)** LC3-II was detected through Western blot. Graph represents the average ± SEM.

To understand the mechanism underlying Ang-(1-9) effect on autophagy, we examined the intracellular signaling pathways triggered by Ang-(1-9) in cardiomyocytes, with a focus on the AT2R/Akt/ERK signaling axis. Early signaling events were probed by exposing cardiomyocytes to 10 μM Ang-(1-9) for intervals ranging from 10 to 60 min, tracking Akt and ERK1/2 phosphorylation via Western blot (Figures 3A,B). Ang-(1-9) elicited a time-dependent elevation in p-Akt and p-ERK1/2, with ERK1/2 activation preceding Akt. Notably, a significant spike in ERK1/2 phosphorylation was detected as early as 10 min, persisting through the 15-min mark. Akt phosphorylation saw an uptick from the 15 to 30-min interval post-Ang-(1-9) treatment. The involvement of AT2R was further confirmed by pre-treating cardiomyocytes with the AT2R antagonist PD123319 (2 μM) prior to Ang-(1-9) exposure, which significantly mitigated the activation of p-Akt and p-ERK1/2 (Figures 3C,D), albeit not entirely for p-ERK1/2, suggesting that ERK activation by Ang-(1-9) also proceeds via alternative pathways (42, 43).

The effect of AT2R inhibition on autophagy, following Ang-(1-9) addition, was also explored. NRVMs cultured with 10 μM Ang-(1-9) in the presence or absence of 2 μM PD123319 (Figure 3E), as well as inhibitors of Akt and ERK1/2, 5 µM Akti and 10 µM UO126 respectively (Supplementary Figure S3), were examined. Confocal imaging of indirect immunofluorescence staining showed that the reduction in LC3 autophagosome puncta, induced by Ang-(1-9), was obstructed by PD123319 treatment (Figure 3E), and basal LC3-II protein levels were restored (Figure 3F). This indicates that the Ang-(1-9)-induced reduction in autophagic flux necessitates AT2R activation. A comparable outcome was observed following Akt and ERK inhibition (Supplementary Figure S3).

In conclusion, our findings reveal that Ang-(1-9) activates the AT2R/Akt/ERK signaling pathway, influencing the autophagic landscape in cardiomyocytes. Blocking AT2R, Akt, and ERK1/2 effectively halts these signaling cascades and the accompanying autophagic alterations, illuminating the regulatory mechanisms at play.

### Role of Ang-(1-9) on the major autophagy agent beclin-1 (BCN1)

Beclin-1 (BCN1) is a key autophagy regulator, acting as an essential initiator of autophagosome formation (44, 45) and as a scaffold for recruiting other autophagy-related proteins and lipids to construct the autophagosome membrane (46–50). We aimed to uncover the effects of Ang-(1-9) on BCN1 expression and its regulation via phosphorylation to understand the underlying regulatory mechanisms.

**Figure 4:**
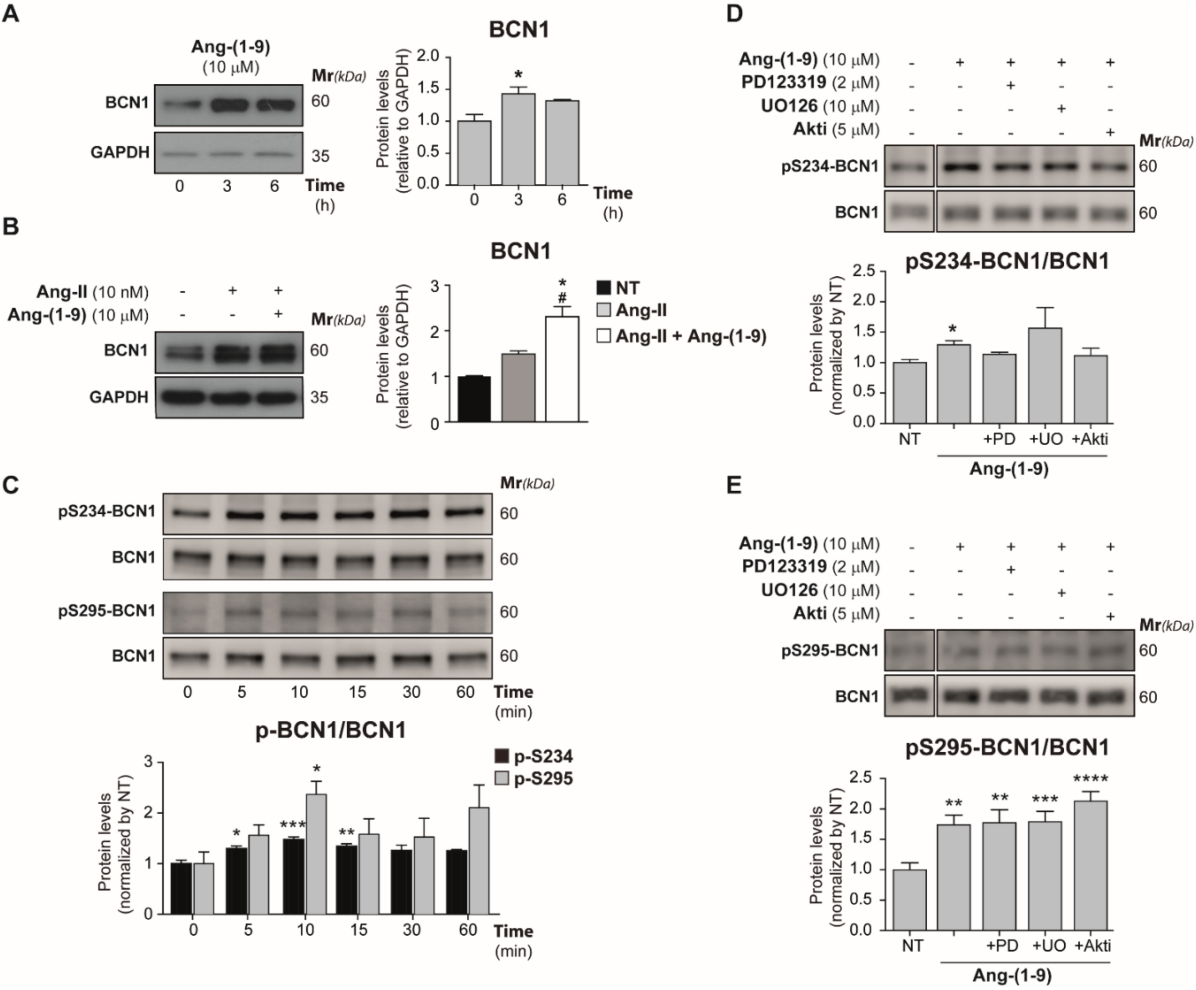
Angiotensin-(1-9) induces BCN1 expression and inhibitory phosphorylations. **(A)** Cardiomyocytes were cultured for 3 and 6 h with 10 μM Ang-(1-9) and levels of BCN1 were detected through Western blot. The graph represents the average ± SEM of *n* = 3. **(B)** Cells were cultured by 24 h with 10 nM Ang-II and 10 μM Ang-(1-9) (the last 6 h) and BCN1 protein levels were detected through Western blot. The graph represents the average ± SEM from at least *n* = 3 of protein levels. Data were relativized to GAPDH levels and normalized by NT condition. **(C)** Cells were treated for 5–60 min with 10 μM Ang-(1-9) and time-dependent phosphorylation of S234 and S295-BCN1 residues were detected by Western blot. The graph represents the average ± SEM of *n* = 4. Cardiomyocytes were treated with 2 μM PD123319 (PD), 5 μM Akti-1/2 (Akti) or 10 μM UO126 (UO) for 30 min before to 10 μM Ang-(1-9) by 10 min to detect through Western-blot the levels of phospho-S234-BCN1 **(D)** and phospho-S295-BCN1 **(E)** Graphs represent the average ± SEM of *n* = 4. *p < 0.05; **p < 0.01; ***p < 0.001 and ****p < 0.0001 condition vs. 0 h or NT. One-way ANOVA and Dunnet's post-test.

We observed that treatment with 10 μM Ang-(1-9) led to elevated BCN1 levels in cardiomyocytes (Figure 4A), both untreated and those previously exposed to Ang-II, indicating a nuanced interaction between Ang-II and Ang-(1-9) in modulating BCN1 expression (Figure 4B). Further investigations into the phosphorylation of BCN1 at serine 234 (p-S234) and serine 295 (p-S295), which are associated with autophagy inhibition (45, 51), revealed dynamic, time-dependent changes in phosphorylation at these sites. Specifically, phosphorylation at p-S234 remained consistent between 5 and 15 min, while p-S295 peaked at 10 min of treatment, suggesting a temporal pattern to BCN1 phosphorylation (Figure 4C).

Finaly, cardiomyocytes were pre-treated with inhibitors 2 μM PD123319, 5 μM Akti, or 10 μM UO126 prior to a 10 min Ang-(1-9) exposure to assess its influence on p-S234 and p-S295 levels (Figures 4D,E). These inhibitors differentially affected BCN1 phosphorylation in response to Ang-(1-9), with p-S234 phosphorylation being reliant on the AT2R, Akt, and ERK1/2 signaling pathways (Figure 4D). In contrast, p-S295 phosphorylation remained unchanged in the presence of these inhibitors, suggesting its independence from the AT2R/Akt/ERK1/2 axis (Figure 4E). These results offer valuable insights into the complex mechanisms underlying Ang-(1-9)-induced cellular responses in cardiomyocytes and emphasize its therapeutic potential.

## Discussion

Maintaining a balanced activation of autophagy is vital for regulating cardiomyocyte function and overall heart physiology. Constitutive autophagy is fundamental for preserving cellular homeostasis within the heart, ensuring the proper functioning and longevity of cardiac cells (21). Any disruption in this equilibrium is linked to a variety of cardiovascular diseases, such as coronary artery disease and heart failure (22, 52, 53).

The relationship between autophagy and cardiac hypertrophy is complex, with evidence suggesting that increased autophagy can lead to either pathological hypertrophy or a reversion of hypertrophy. Under physiological conditions, autophagy supports cardiac function by removing damaged organelles and misfolded proteins (54, 55). However, the activation of autophagy in pathological conditions such as pressure overload or neurohumoral stimuli shows divergent outcomes. On one hand, studies indicate that enhanced autophagic activity can mitigate the development of cardiac hypertrophy. For instance, Zhao et al. demonstrated that promoting autophagy through the AKT/mTOR pathway can inhibit pressure overload-induced cardiac hypertrophy, suggesting a protective role for autophagy in this context (56). Furthermore, autophagy is protective during the transition from hypertrophy to heart failure (57). Conversely, excessive autophagy can lead to detrimental effects, including cell death and impaired cardiac function. For example, studies have reported that excessive autophagic flux can exacerbate cardiac hypertrophy and contribute to heart failure (58, 59). Similarly, research by Cao et al. highlighted that overexpression of BCN1, an essential autophagy regulator, can lead to maladaptive hypertrophic growth, which can be antagonized by a histone deacetylase inhibitor, indicating that a finely tuned autophagic response is necessary to prevent pathological remodeling in response to cardiac overload (60). This paradoxical effect is summarized by Li et al., who noted that while autophagy is crucial for maintaining cardiac homeostasis, its dysregulation can promote pathological hypertrophy (61). The balance between sufficient and excessive autophagy is critical; while moderate activation may prevent hypertrophy, excessive autophagy can lead to cellular dysfunction and hypertrophic progression (62).

LC3-II is a crucial protein in the autophagy pathway, essential for autophagosome formation. During autophagy induction, LC3-I undergoes lipidation to LC3-II, attaching itself to the autophagosomal membrane. Concurrently, p62, serving as a selective autophagy receptor, binds and directs cellular constituents towards degradation. Typically, as autophagy progresses, p62 levels decrease as it is consumed along with the targeted cargo within lysosomes. Therefore, a rise in LC3-II alongside a fall in p62 levels often signals active autophagy, indicative of increased autophagosome formation and enhanced degradation of cellular components (63).

Since autophagy is a dynamic and continuous process involving not only autophagosome formation but also the subsequent steps of maturation, fusion with lysosomes, and degradation, we evaluated the autophagic markers in the presence of the autophagic flux blocker CQ. This strategy revealed that Ang-(1-9) reduces basal autophagy, evidenced by the increase of LC3-II and the reduction of p62 levels.

To our knowledge, this is the first time that Ang-(1-9) has been linked to the regulation of autophagy, joining other members of the RAS involved in the control of this catabolic process, such as Ang-(1-7) (64, 65) and Ang-II (41). The RAS is widely known for its important role in the onset and progression of heart failure (4, 66, 67). In the canonical pathway, ACE generates Ang-II from Ang-I, exerting deleterious effects on the myocardium and vasculature through the AT1R (68, 69). Conversely, ACE2 generates Ang-(1-7) from Ang-II (66, 67, 70, 71) and Ang-(1-9) from Ang-I (7).

Ang-(1-7) and Ang-(1-9) act through the Mas receptor (MasR) or the AT2R, respectively, to antagonize the harmful actions of Ang-II, stimulating vasodilation and anti-inflammatory responses (72, 73). Previous research has demonstrated that the autophagy process can be influenced by the RAS receptors, specifically AT1R and AT2R. Notably, activation of the AT2R receptor is thought to enhance autophagy across various tissues, a mechanism critical for cell health and longevity (29, 33, 42, 74–77). In our study, we found that Ang-(1-9) acting through AT2R, not only reduces basal autophagy in cardiomyocytes but also modulates autophagy levels in response to Ang-II exposure (Figure 2). This observation highlights the complex role of RAS in regulating cardiovascular physiology and underscores the importance of AT2R in the autophagic pathway.

Ang-II is known to activate autophagy through the AT1R across various cell types, including cardiomyocytes and podocytes, highlighting its broad impact (33, 78). Specifically, in the context of cardiac health, AT1R plays a crucial role during myocardial ischemia/reperfusion incidents, engaging signaling pathways like p38-MAPK and reactive oxygen species (ROS) signaling to regulate autophagy (34, 79–82). Moreover, Ang-II-induced autophagy in cardiomyocytes engages the PI3K-Akt-mTOR signaling pathway (83). Interestingly, the inhibition of miR-128 activates the PIK3R1/Akt/mTOR pathway, thereby countering the autophagy induced by Ang-II (84). Additionally, the Ang-(1-7)/MasR signaling axis can also inhibit Ang-II-induced autophagy by reducing ROS levels (81). Ang 1-7 also exhibits strong anti-inflammatory and anti-fibrotic properties, playing a crucial role in mitigating cardiac remodeling, hypertrophy, and fibrosis associated with hypertension and heart failure, and reduces thrombosis and vascular calcification demonstrating protective effects against atherosclerosis and aneurysm formation in preclinical models (85, 86).

Conversely, the AT2R exerts an opposing effect on autophagy within cardiomyocytes. Activation of AT2R leads to autophagy inhibition, a response that contrasts with the autophagy induction seen with AT1R (33). This inhibition of autophagy was shown in a rat model of heart hypertrophy, suggesting the involvement of a PI3K-dependent signaling mechanism (87, 88). Ang-(1-9), which predominantly interacts with AT2R, has been shown to significantly reduce cardiac fibrosis and prevent cardiomyocyte hypertrophy. The underlying mechanisms likely involve the regulation of mitochondrial dynamics and intracellular calcium levels, as well as the inhibition of the calcineurin/NFAT signaling pathway (17, 89).

Our study advances the understanding of the complex interplay between AT2R and Ang-II-induced autophagy. This work sheds light on the sophisticated regulatory networks influencing cardiovascular health and disease. Notably, our research pioneers in identifying a link between Ang-(1-9) and autophagy regulation via AT2R, mediated through the Akt/ERK signaling pathway. This breakthrough marks a significant step forward in understanding the complex roles of angiotensin derivatives in cardiomyocyte biology.

Our results show that Ang-(1-9) inhibits cardiac hypertrophy despite activating the Akt2/mTOR pathway, a signal typically linked to cardioprotection, but also with increased protein synthesis and hypertrophy. Akt, particularly the Akt2 isoform, is integral to cellular signaling and has been associated with either promoting or inhibiting cardiac hypertrophy. While the activation of the Akt pathway can lead to hypertrophic responses in cardiomyocytes via the mTOR signaling pathway (90), Akt2-deficient mice exhibited normal cardiac morphology and function, but a trend to develop spontaneous hypertrophy with aging and increased hypertrophic response to isoproterenol compared with wild type mice (91). Moreover, the three Akt kinase isoforms (Akt1, Akt2, and Akt3) uniquely influence cardiac responses to different stimuli. For instance, Akt1-KO mice resist hypertrophy from swimming training but show enhanced hypertrophy following 7 days of TAC surgery (92). In contrast, while WT and Akt2-KO mice have similar responses to TAC, Akt2-KO mice experience larger infarcts after permanent LAD occlusion at 7 days due to higher apoptosis rates, despite initial infarct sizes being comparable at 24 h (92, 93). The role of Akt is further complicated by the involvement of other signaling pathways, which can modulate the hypertrophic response (94). These findings suggest that the effect of Akt2 is strongly dependent upon the cellular and physiological context and that under specific conditions, Akt2 activation may lead to responses different from cardiac hypertrophy.

The evidence supporting the activation of the Akt/ERK pathway by Ang-(1-9) is limited but consistent. For instance, Mendoza-Torres et al. observed an AT2R/Akt pathway activation after treatment with Ang-(1-9) in isolated rat hearts during reperfusion, which promotes a reduction in cell death, an improvement of left ventricle function after myocardial infarction, and a decrease in infarct size. The inhibition of Akt blocked the cardioprotection given by Ang-(1-9) both *in vitro* and *ex vivo* (15). These findings are consistent with our results and other studies showing that AT2R mediates Akt activation (20, 95) and supports the idea that Ang-(1-9) could play the counter-regulatory to Ang-II and protective heart effect through AT2R and Akt regulating the autophagy activation. The participation of ERK has also been observed in rats with adriamycin-induced cardiomyopathy where Ang-(1-9) improves left ventricular function through an AT2R/ERK1/2 and p38MAPK-dependent mechanism (96). An alternative explanation that may contribute to the beneficial effect of Ang-(1-9) on autophagy depends on its ability to increase ACE2 levels and consequently decrease Ang-II levels, thus reducing basal autophagy levels (97).

To further study the mechanism through which Ang-(1-9) regulates autophagy, we evaluated BCN1, a key regulator in the initial steps of autophagy and a target of multiple post-translational control (51). BCN1 has a pivotal function in maintaining cellular homeostasis, and its involvement is extensively documented across a spectrum of diseases, including cancer (98–101), and cardiovascular disorders (102).

Phosphorylation and ubiquitination of BCN1 at multiple residues fine-tune the response to autophagy-modulating stimuli and help in maintaining the balance between pro-survival autophagy and pro-apoptotic responses (103, 104). Phosphorylation of BCN1 can be produced by diverse protein kinases, including ULK1 (105, 106), CAMKII (107), AMPK (108, 109), Akt (45) and FAK (110). BCN1 phosphorylation can enhance or inhibit its activity, depending on the specific phosphorylation sites and the kinases involved, as the S234 and S295 phosphorylations Akt-mediated evaluated in this work (45). The precise phosphorylation pattern can be context-dependent and is critical in modulating autophagy in response to various cellular signals, including nutrient availability, stress, and growth factor signaling (51).

BCN1 is characterized by three major functional domains that play critical roles in autophagy: a) Intrinsically disordered N-terminal region and BCL-2 homology domain (111): The dissociation of BCN1 from BCL-2/BCL-XL proteins is crucial for initiating autophagy (44, 103, 112). This interaction, importantly, does not influence the anti-apoptotic functions of BCL-2/BCL-XL (113, 114); b) Coiled-coil domain (CCD): This domain enables BCN1 to interact with either UVRAG or ATG14, leading to the formation of two distinct class III PI3K complexes: Complex 1 (C1) and Complex 2 (C2). Within these complexes, the catalytic lipid kinase subunit VSP34 phosphorylates phosphatidylinositol, crucial for autophagy and membrane trafficking (115). The phosphorylation site S234 within this domain (residues 174–266) impairs BCN1's ability to form C1 or C2 complexes (116). It suggests that the negative regulation of autophagy by Ang-(1-9) could be due to increased S234 phosphorylation, which inhibits the formation of these complexes. However, this hypothesis was not explored in our study; c) β-α Autophagy-specific (BARA) Domain (residues 266–450): This domain interacts with membranes (48). Located within this domain, the inhibitory phosphorylation at S295 could affect BCN1's membrane association (48, 50, 115). This, in combination with S234 phosphorylation, may alter BCN1's subcellular localization and function, a topic for future investigation.

**Figure 5:**
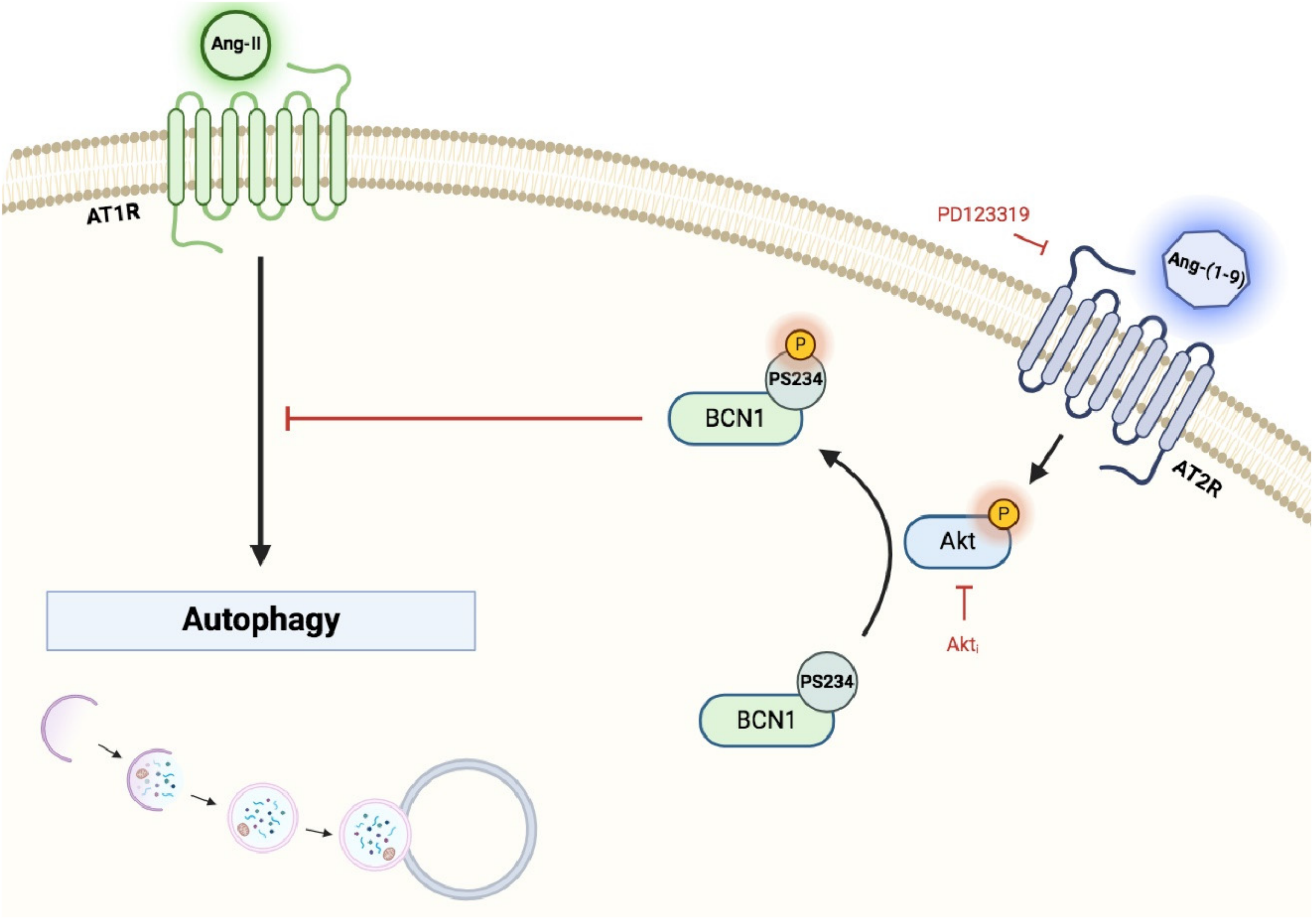
Summary scheme. Ang-(1-9) triggers Akt activation via AT2R and leads the phosphorylation of BCN1 at S234 and S295 which in turn reduces the autophagy levels.

Figure 5 provides a summary of our findings. In our model, Ang-(1-9) triggers Akt activation via AT2R. This activation leads to the phosphorylation of BCN1 at S234 and S295, which in turn reduces autophagy levels (45, 108, 117). The phosphorylation by Akt enhances BCN1's association with the adaptor protein 14-3-3 and the intermediate filament vimentin, anchoring it to the cytoskeleton and thereby inhibiting autophagy (45). This interaction may interfere with the cell's ability to respond to other pro-autophagic signals. Notably, within the context of chemotherapy, phosphorylation at BCN1's S234/S295 has been observed to promote caspase activation, leading to increased apoptosis and further suppression of autophagy (118). While the interplay between apoptosis and autophagy in our model presents an area for future investigation, these findings contribute to a deeper understanding of the complex mechanisms by which Ang-(1-9) influences cellular processes, laying the groundwork for further exploration into its role in cardiomyocyte biology and beyond. Additionally, our study provides valuable insights into the role of Ang-(1-9) in autophagy regulation through *in vitro* experiments, we recognize the importance of complementing these findings with animal studies. Future investigations using *in vivo* models will be essential to further validate our results and to explore the wider physiological and therapeutic implications of Ang-(1-9) in cardiovascular health.

## Conclusions

The results presented in this work show that basal autophagy is inhibited in cardiomyocytes by action of Ang-(1-9) through AT2R, and the kinase Akt.

Besides, Ang II-induced autophagy is restrained in presence of Ang-(1-9), This reduction unveils a novel aspect of Ang-(1-9) action, that potentially contributing to cardioprotective effects of this nonapeptide.

## Data Availability

The original contributions presented in the study are included in the article/Supplementary Material, further inquiries can be directed to the corresponding authors.

## Supplementary material

Supplementary Figure S1 **(A)** Confocal images of indirect immunofluorescence and detection of autophagosome's LC3 dot pattern (green), and nuclei staining (blue, Hoescht 33342) in cardiomyocytes cultured by 24 h with 10 μM Ang-(1-9). Scale bar = 2 μm. Cardiomyocytes were cultured by 24 h in the presence or absence of 10 μM Ang-(1-9) and 50 nM Baf-A (the last 6 h) and levels of LC3-II **(B)** and p62 **(C)** were determined through Western-blot and quantified. The graphs represent the average ± SEM of *n* = 3.

Supplementary Figure S2 Cardiomyocytes were cultured by 24 h in the presence of different concentrations of Ang-II (10–500 nM) and 50 nM Baf-A (the last 6 h) and levels of LC3-II were determined through Western-blot and quantified. The graph represents the average ± SEM of *n* = 3 of data in Baf-A presence. **p* < 0.05 condition vs. Baf-A. One-way ANOVA and Dunnet's post-test.

Supplementary Figure S3 Confocal images of indirect immunofluorescence and detection of autophagosome's LC3 dot pattern (green), actin cytoskeleton (red, phalloidin), and nuclei staining (blue, Hoescht 33342) in cardiomyocytes cultured by 24 h with 10 mM Ang-(1-9) in the presence or absence of 5 mM Akti-1/2 (Akti) or 10 mM UO126. The cells were pre-incubated during 30 min before the stimuli with the respective inhibitors. Scale bar = 2 μm. NT, non-treated.

## References

[1] Ferrario CM. Cardiac remodelling and RAS inhibition. Ther Adv Cardiovasc Dis. (2016) 10(3):162–71. 10.1177/1753944716642677 27105891 PMC5192558

[2] Ocaranza MP Riquelme JA García L Jalil JE Chiong M Santos RAS Counter-regulatory renin-angiotensin system in cardiovascular disease. Nat Rev Cardiol. (2020) 17(2):116–29. 10.1038/s41569-019-0244-8 31427727 PMC7097090

[3] Poznyak AV Bharadwaj D Prasad G Grechko AV Sazonova MA Orekhov AN. Renin-angiotensin system in pathogenesis of atherosclerosis and treatment of cvd. Int J Mol Sci. (2021) 22(13):6702. 10.3390/ijms22136702 34206708 PMC8269397

[4] Mehta JK Kaur G Buttar HS Bagabir HA Bagabir RA Bagabir SA Role of the renin-angiotensin system in the pathophysiology of coronary heart disease and heart failure: diagnostic biomarkers and therapy with drugs and natural products. Front Physiol. (2023) 14:1034170. 10.3389/fphys.2023.1034170 36909245 PMC9995912

[5] Weber KT Sun Y Bhattacharya SK Ahokas RA Gerling IC. Myofibroblast-mediated mechanisms of pathological remodelling of the heart. Nat Rev Cardiol. (2013) 10(1):15–26. 10.1038/nrcardio.2012.158 23207731

[6] Ocaranza MP Jalil JE. Protective role of the ACE2/ang-(19) axis in cardiovascular remodeling. Int J Hypertens. (2012) 2012:594361. 10.1155/2012/594361 22315665 PMC3270559

[7] Donoghue M Hsieh F Baronas E Godbout K Gosselin M Stagliano N A novel angiotensin-converting enzyme-related carboxypeptidase (ACE2) converts angiotensin I to angiotensin 1-9. Circ Res. (2000) 87(5):1–9. 10.1161/01.RES.87.5.e1 10969042

[8] Ocaranza MP Michea L Chiong M Lagos CF Lavandero S Jalil JE. Recent insights and therapeutic perspectives of angiotensin-(1-9) in the cardiovascular system. Clin Sci. (2014) 127(9):549–57. 10.1042/CS20130449 25029123

[9] Caputo I Bertoldi G Driussi G Cacciapuoti M Calò LA. The RAAS goodfellas in cardiovascular system. J Clin Med. (2023) 12(21):6873. 10.3390/jcm12216873 37959338 PMC10649249

[10] Westermeier F Bustamante M Pavez M García L Chiong M Ocaranza MP Novel players in cardioprotection: insulin like growth factor-1, angiotensin-(1–7) and angiotensin-(1–9). Pharmacol Res. (2015) 101:41–55. 10.1016/j.phrs.2015.06.018 26238180

[11] Santos RAS Ferreira AJ Verano-Braga T Bader M. Angiotensin-converting enzyme 2, angiotensin-(1-7) and Mas: new players of the renin-angiotensin system. J Endocrinol. (2013) 216(2):R1–17. 10.1530/JOE-12-0341 23092879

[12] Ocaranza MP Godoy I Jalil JE Varas M Collantes P Pinto M Enalapril attenuates downregulation of angiotensin-converting enzyme 2 in the late phase of ventricular dysfunction in myocardial infarcted rat. Hypertension. (2006) 48(4):572–8. 10.1161/01.HYP.0000237862.94083.45 16908757

[13] Ocaranza MP Lavandero S Jalil JE Moya J Pinto M Novoa U Angiotensin-(1-9) regulates cardiac hypertrophy *in vivo* and *in vitro*. J Hypertens. (2010) 28(5):1054–64. 10.1097/HJH.0b013e328335d291 20411619

[14] Ocaranza MP Moya J Barrientos V Alzamora R Hevia D Morales C Angiotensin-(1-9) reverses experimental hypertension and cardiovascular damage by inhibition of the angiotensin converting enzyme/Ang II axis. J Hypertens. (2014) 32(4):771–83. 10.1097/HJH.0000000000000094 24463937

[15] Mendoza-Torres E Riquelme JA Vielma A Sagredo AR Gabrielli L Bravo-Sagua R Protection of the myocardium against ischemia/reperfusion injury by angiotensin-(1-9) through an AT 2 R and akt-dependent mechanism. Pharmacol Res. (2018) 135:112–21. 10.1016/j.phrs.2018.07.022 30048754

[16] González A Schelbert EB Díez J Butler J. Myocardial interstitial fibrosis in heart failure. J Am Coll Cardiol. (2018) 71(15):1696–706. 10.1016/j.jacc.2018.02.021 29650126

[17] Flores-Munoz M Work LM Douglas K Denby L Dominiczak AF Graham D Angiotensin-(1-9) attenuates cardiac fibrosis in the stroke-prone spontaneously hypertensive rat via the angiotensin type 2 receptor. Hypertension. (2012) 59(2):300–7. 10.1161/HYPERTENSIONAHA.111.177485 22184331

[18] Zheng H Pu SY Fan XF Li XS Zhang Y Yuan J Treatment with angiotensin-(1-9) alleviates the cardiomyopathy in streptozotocin-induced diabetic rats. Biochem Pharmacol. (2015) 95(1):38–45. 10.1016/j.bcp.2015.03.009 25801006

[19] Flores-Muñoz M Smith NJ Haggerty C Milligan G Nicklin SA. Angiotensin1-9 antagonises pro-hypertrophic signalling in cardiomyocytes via the angiotensin type 2 receptor. J Physiol. (2011) 589(4):939–51. 10.1113/jphysiol.2010.203075 21173078 PMC3060371

[20] Cha SA Park BM Gao S Kim SH. Stimulation of ANP by angiotensin- (1-9) via the angiotensin type 2 receptor. Life Sci. (2013) 93(24):934–40. 10.1016/j.lfs.2013.10.020 24177599

[21] Liu SZ Yao SJ Yang H Liu SJ Wang YJ. Autophagy: regulator of cell death. Cell Death Dis. (2023) 14(10):648. 10.1038/s41419-023-06154-8 37794028 PMC10551038

[22] Sciarretta S Maejima Y Zablocki D Sadoshima J. The role of autophagy in the heart. Annu Rev Physiol. (2018) 80:1–26. 10.1146/annurev-physiol-021317-121427 29068766

[23] Orogo AM Gustafsson ÅB. Critical review cell death in the myocardium: my heart won’t go on. IUBMB Life. (2013):651–6. 10.1002/iub.1180 23824949 PMC4074399

[24] Zhou L Ma B Han X. The role of autophagy in angiotensin II-induced pathological cardiac hypertrophy. J Mol Endocrinol. (2016) 57(4):R143–52. 10.1530/JME-16-0086 27620875

[25] Riquelme JA Chavez MN Mondaca-Ruff D Bustamante M Vicencio JM Quest AFG Therapeutic targeting of autophagy in myocardial infarction and heart failure. Expert Rev Cardiovasc Ther. (2016) 14(9):1007–19. 10.1080/14779072.2016.1202760 27308848

[26] Gatica D Chiong M Lavandero S Klionsky DJ. Molecular mechanisms of autophagy in the cardiovascular system. Circ Res. (2015) 116(3):456–67. 10.1161/CIRCRESAHA.114.303788 25634969 PMC4313620

[27] Lavandero S Troncoso R Rotherme BA Martinet W Sadoshima J Hill JA. Cardiovascular autophagy concepts, controversies, and perspectives. Autophagy. (2013) 9(10):1455–66. 10.4161/auto.25969 23959233

[28] Aghaei M Motallebnezhad M Ghorghanlu S Jabbari A Enayati A Rajaei M Targeting autophagy in cardiac ischemia/reperfusion injury: a novel therapeutic strategy. J Cell Physiol. (2019) 234(10):16768–78. 10.1002/jcp.28345 30807647

[29] Porrello ER Delbridge LMD. Cardiomyocyte autophagy is regulated by angiotensin II type 1 and type 2 receptors. Autophagy. (2009) 5(8):1215–6. 10.4161/auto.5.8.10153 19955853

[30] Silva KAS Ghiarone T Schreiber K Grant DA White T Frisard MI Angiotensin II suppresses autophagy and disrupts ultrastructural morphology and function of mitochondria in mouse skeletal muscle. J Appl Physiol. (2019) 126(6):1550–62. 10.1152/japplphysiol.00898.2018 30946636 PMC6620657

[31] Dai DF Rabinovitch P. Mitochondrial oxidative stress mediates induction of autophagy and hypertrophy in angiotensin-II treated mouse hearts. Autophagy. (2011) 7(8):917–8. 10.4161/auto.7.8.15813 21505274 PMC3359471

[32] Duan H Li Y Yan L Yang H Wu J Qian P Rcan1-1l overexpression induces mitochondrial autophagy and improves cell survival in angiotensin II-exposed cardiomyocytes. Exp Cell Res. (2015) 335(1):99–106. 10.1016/j.yexcr.2015.05.003 25978972

[33] Porrello ER D’Amore A Curl CL Allen AM Harrap SB Thomas WG Angiotensin II type 2 receptor antagonizes angiotensin ii type 1 receptor-mediated cardiomyocyte autophagy. Hypertension. (2009) 53(6):1032–40. 10.1161/HYPERTENSIONAHA.108.128488 19433781

[34] Xiao R Zhao HC Yan TT Zhang Q Huang YS. Angiotensin II and hypoxia induce autophagy in cardiomyocytes via activating specific protein kinase C subtypes. Cardiovasc Diagn Ther. (2021) 11(3):744–59. 10.21037/cdt-20-883 34295701 PMC8261756

[35] Galvez A Morales MP Eltit JM Ocaranza P Carrasco L Campos X A rapid and strong apoptotic process is triggered by hyperosmotic stress in cultured rat cardiac myocytes. Cell Tissue Res. (2001) 304(2):279–85. 10.1007/s004410100358 11396721

[36] Pfaffl MW. A new mathematical model for relative quantification in real-time RT-PCR. Nucleic Acids Res. (2001) 29(9):45e–45. 10.1093/nar/29.9.e45 PMC55695 11328886

[37] Vesentini N Barsanti C Martino A Kusmic C Ripoli A Rossi A Selection of reference genes in different myocardial regions of an *in vivo* ischemia/reperfusion rat model for normalization of antioxidant gene expression. BMC Res Notes. (2012) 5(1):124. 10.1186/1756-0500-5-124 22377061 PMC3392735

[38] Zhang M Xiao X Xiong D Liu Q. Topic-based dissimilarity and sensitivity models for translation rule selection. J Artif Intell Res. (2014) 50:1–30. 10.1613/jair.4265

[39] Mauthe M Orhon I Rocchi C Zhou X Luhr M Hijlkema KJ Chloroquine inhibits autophagic flux by decreasing autophagosome-lysosome fusion. Autophagy. (2018) 14(8):1435–55. 10.1080/15548627.2018.1474314 29940786 PMC6103682

[40] Mondaca-Ruff D Riquelme JA Quiroga C Norambuena-Soto I Sanhueza-Olivares F Villar-Fincheira P Angiotensin II-regulated autophagy is required for vascular smooth muscle cell hypertrophy. Front Pharmacol. (2019) 9:1–13. 10.3389/fphar.2018.01553 PMC6371839 30804791

[41] Kobara M Toba H Nakata T. Roles of autophagy in angiotensin II-induced cardiomyocyte apoptosis. Clin Exp Pharmacol Physiol. (2022) 49(12):1342–51. 10.1111/1440-1681.13719 36059129

[42] Hassani B Attar Z Firouzabadi N. The renin-angiotensin-aldosterone system (RAAS) signaling pathways and cancer: foes versus allies. Cancer Cell Int. (2023) 23(1):1–27. 10.1186/s12935-023-03080-9 37891636 PMC10604988

[43] Kong T Liu M Ji B Bai B Cheng B Wang C. Role of the extracellular signal-regulated kinase 1/2 signaling pathway in ischemia-reperfusion injury. Front Physiol. (2019) 10:1038. 10.3389/fphys.2019.01038 31474876 PMC6702336

[44] Maiuri MC Criollo A Tasdemir E Vicencio JM Tajeddine N Hickman JA BH3-only proteins and BH3 mimetics induce autophagy by competitively disrupting the interaction between beclin 1 and bcl-2/bcl-XL. Autophagy. (2007) 3(4):374–6. 10.4161/auto.4237 17438366

[45] Wang RC Wei Y An Z Zou Z Xiao G Bhagat G Akt-mediated regulation of autophagy and tumorigenesis through beclin 1 phosphorylation. Science (80-). (2012) 338(6109):956–9. 10.1126/science.1225967 PMC3507442 23112296

[46] Huang W Choi W Hu W Mi N Guo Q Ma M Crystal structure and biochemical analyses reveal beclin 1 as a novel membrane binding protein. Cell Res. (2012) 22(3):473–89. 10.1038/cr.2012.24 22310240 PMC3292424

[47] Rostislavleva K Soler N Ohashi Y Zhang L Pardon E Burke JE Structure and flexibility of the endosomal Vps34 complex reveals the basis of its function on membranes. Science (80-). (2015) 350(6257):1–25. 10.1126/science.aac7365 PMC4601532 26450213

[48] Noda NN Kobayashi T Adachi W Fujioka Y Ohsumi Y Inagaki F. Structure of the novel C-terminal domain of vacuolar protein sorting 30/autophagy-related protein 6 and its specific role in autophagy. J Biol Chem. (2012) 287(20):16256–66. 10.1074/jbc.M112.348250 22437838 PMC3351336

[49] Stjepanovic G Baskaran S Lin MG Hurley JH. Vps34 kinase domain dynamics regulate the autophagic PI 3-kinase Complex. Mol Cell. (2017) 67(3):528–34.e3. 10.1016/j.molcel.2017.07.003 28757208 PMC5573195

[50] Chang C Young LN Morris KL von Bülow S Schöneberg J Yamamoto-Imoto H Bidirectional control of autophagy by BECN1 BARA domain dynamics. Mol Cell. (2019) 73(2):339–53.e6. 10.1016/j.molcel.2018.10.035 30581147 PMC6450660

[51] Menon MB Dhamija S. Beclin 1 phosphorylation—at the center of autophagy regulation. Front Cell Dev Biol. (2018) 6:1–9. 10.3389/fcell.2018.00137 30370269 PMC6194997

[52] Popov SV Mukhomedzyanov AV Voronkov NS Derkachev IA Boshchenko AA Fu F Regulation of autophagy of the heart in ischemia and reperfusion. Apoptosis. (2023) 28(1–2):55–80. 10.1007/s10495-022-01786-1 36369366

[53] Mialet-Perez J Vindis C. Autophagy in health and disease: focus on the cardiovascular system. Essays Biochem. (2017) 61(6):721–32. 10.1042/EBC20170022 29233881

[54] Baba SP Zhang D Singh M Dassanayaka S Xie Z Jagatheesan G Deficiency of aldose reductase exacerbates early pressure overload-induced cardiac dysfunction and autophagy in mice. J Mol Cell Cardiol. (2018) 118:183–92. 10.1016/j.yjmcc.2018.04.002 29627295 PMC6205513

[55] Yuan S Jin J Chen L Hou Y Wang H. Naoxintong/PPAR γ signaling inhibits cardiac hypertrophy via activation of autophagy. Evid Based Complement Alternat Med. (2017) 2017:3801976. 10.1155/2017/3801976 28293264 PMC5331281

[56] Zhao D Wang W Wang H Peng H Liu X Guo W PKD knockdown inhibits pressure overload-induced cardiac hypertrophy by promoting autophagy via AKT/mTOR pathway. Int J Biol Sci. (2017) 13(3):276–85. 10.7150/ijbs.17617 28367092 PMC5370435

[57] Wang ZP Shen D Che Y Jin YG Wang SS Wu QQ Corosolic acid ameliorates cardiac hypertrophy via regulating autophagy. Biosci Rep. (2019) 39(12):1–14. 10.1042/BSR20191860 PMC6893168 31746323

[58] Lin L Liu X Xu J Weng L Ren J Ge J High-density lipoprotein inhibits mechanical stress-induced cardiomyocyte autophagy and cardiac hypertrophy through angiotensin II type 1 receptor-mediated PI3K/akt pathway. J Cell Mol Med. (2015) 19(8):1929–38. 10.1111/jcmm.12567 25946687 PMC4549043

[59] Qi H Ren J Ba L Song C Zhang Q Cao Y MSTN attenuates cardiac hypertrophy through inhibition of excessive cardiac autophagy by blocking AMPK/mTOR and miR-128/PPARγ/NF-κB. Mol Ther Nucleic Acids. (2020) 19:507–22. 10.1016/j.omtn.2019.12.003 31923740 PMC6951838

[60] Cao DJ Wang ZV Battiprolu PK Jiang N Morales CR Kong Y Histone deacetylase (HDAC) inhibitors attenuate cardiac hypertrophy by suppressing autophagy. Proc Natl Acad Sci U S A. (2011) 108(10):4123–8. 10.1073/pnas.1015081108 21367693 PMC3053983

[61] Li L Xu J He L Peng L Zhong Q Chen L The role of autophagy in cardiac hypertrophy. Acta Biochim Biophys Sin (Shanghai). (2016) 48(6):491–500. 10.1093/abbs/gmw025 27084518 PMC4913516

[62] Ren S Shen L Lin S Xiao D Xiao W Yan PM Mechanistic analysis of resveratrol in cardiac hypertrophy by network pharmacology and animal experiments. Mol Med Rep. (2022) 26(5):1–11. 10.3892/mmr.2022.12840 PMC9727583 36052855

[63] Klionsky DJ Abdel-Aziz AK Abdelfatah S Abdellatif M Abdoli A Abel S Guidelines for the use and interpretation of assays for monitoring autophagy (4th edition)^1^. Autophagy. (2021) 17(1):1–382. 10.1080/15548627.2020.1797280 33634751 PMC7996087

[64] Rivera JC Abrigo J Tacchi F Simon F Brandan E Santos RA Angiotensin-(1-7) prevents lipopolysaccharide-induced autophagy via the mas receptor in skeletal muscle. Int J Mol Sci. (2020) 21(24):1–18. 10.3390/ijms21249344 PMC7762589 33302427

[65] Jiang T Gao L Zhu XC Yu JT Shi JQ Tan MS Angiotensin-(1-7) inhibits autophagy in the brain of spontaneously hypertensive rats. Pharmacol Res. (2013) 71:61–8. 10.1016/j.phrs.2013.03.001 23499735

[66] Yang B Li D Phillips MI Mehta P Mehta JL. Myocardial angiotensin II receptor expression and ischemia-reperfusion injury. Vasc Med. (1998) 3(2):121–30. 10.1177/1358836X9800300206 9796075

[67] Paul M Mehr AP Kreutz R. Physiology of local renin-angiotensin systems. Physiol Rev. (2006) 86(3):747–803. 10.1152/physrev.00036.2005 16816138

[68] Raizada MK Ferreira AJ. ACE2: a new target for cardiovascular disease therapeutics. J Cardiovasc Pharmacol. (2007) 50(2):112–9. 10.1097/FJC.0b013e3180986219 17703127

[69] Mendoza Torres E Oyarzún A Mondaca Ruff D Azocar A Chiong M Castro PF ACE2 and vasoactive peptides: novel players in cardiovascular/renal remodeling and hypertension. Ther Adv Cardiovasc Dis. (2015) 9(4):217–37. 10.1177/1753944715597623 26275770

[70] Roth GA Huffman MD Moran AE Feigin V Mensah GA Naghavi M Global and regional patterns in cardiovascular mortality from 1990 to 2013. Circulation. (2015) 132(17):1667–78. 10.1161/CIRCULATIONAHA.114.008720 26503749

[71] Hausenloy DJ Yellon DM. Ischaemic conditioning and reperfusion injury. Nat Rev Cardiol. (2016) 13(4):193–209. 10.1038/nrcardio.2016.5 26843289

[72] Patel SN Fatima N Ali R Hussain T. Emerging role of angiotensin AT2 receptor in anti-inflammation: an update. Curr Pharm Des. (2020) 26(4):492–500. 10.2174/1381612826666200115092015 31939729 PMC7457547

[73] Dhande I Ma W Hussain T. Angiotensin AT2 receptor stimulation is anti-inflammatory in lipopolysaccharide-activated THP-1 macrophages via increased interleukin-10 production. Hypertens Res. (2015) 38(1):21–9. 10.1038/hr.2014.132 25209104 PMC4898267

[74] Qi Y Li H Shenoy V Li Q Wong F Zhang L Moderate cardiac-selective overexpression of angiotensin II type 2 receptor protects cardiac functions from ischaemic injury. Exp Physiol. (2012) 97(1):89–101. 10.1113/expphysiol.2011.060673 21967903 PMC3619662

[75] Sun L Wang W Xiao W Liang H Yang Y Yang H. Angiotensin II induces apoptosis in intestinal epithelial cells through the AT2 receptor, GATA-6 and the bax pathway. Biochem Biophys Res Commun. (2012) 424(4):663–8. 10.1016/j.bbrc.2012.07.003 22776205

[76] Pickel L Matsuzuka T Doi C Ayuzawa R Maurya DK Xie SX Overexpression of angiotensin II type 2 receptor gene induces cell death in lung adenocarcinoma cells. Cancer Biol Ther. (2010) 9(4):277–85. 10.4161/cbt.9.4.10643 20026904 PMC2974059

[77] Kawabata A Baoum A Ohta N Jacquez S Seo GM Berkland C Intratracheal administration of a nanoparticle-based therapy with the angiotensin II type 2 receptor gene attenuates lung cancer growth. Cancer Res. (2012) 72(8):2057–67. 10.1158/0008-5472.CAN-11-3634 22389453 PMC3566878

[78] Bensaada I Robin B Perez J Salemkour Y Chipont A Camus M Calpastatin prevents angiotensin II–mediated podocyte injury through maintenance of autophagy. Kidney Int. (2021) 100(1):90–106. 10.1016/j.kint.2021.02.024 33675847

[79] Dai W Hale SL Kay GL Jyrala AJ Kloner RA. Cardioprotective effects of angiotensin ii type 1 receptor blockade with olmesartan on reperfusion injury in a rat myocardial ischemia-reperfusion model. Cardiovasc Ther. (2010) 28(1):30–7. 10.1111/j.1755-5922.2009.00108.x 20074257

[80] Lin L Tang C Xu J Ye Y Weng L Wei W Mechanical stress triggers cardiomyocyte autophagy through angiotensin II type 1 receptor-mediated p38MAP kinase independently of angiotensin II. PLoS One. (2014) 9(2):1–8. 10.1371/journal.pone.0089629 PMC3931796 24586922

[81] Lin L Liu X Xu J Weng L Ren J Ge J Mas receptor mediates cardioprotection of angiotensin-(1-7) against angiotensin II-induced cardiomyocyte autophagy and cardiac remodelling through inhibition of oxidative stress. J Cell Mol Med. (2016) 20(1):48–57. 10.1111/jcmm.12687 26515045 PMC4717848

[82] Dai D-F Johnson SC Villarin JJ Chin MT Nieves-Cintrón M Chen T Mitochondrial oxidative stress mediates angiotensin II-induced cardiac hypertrophy and galphaq overexpression-induced heart failure. Circ Res. (2011) 108(7):837–46. 10.1161/CIRCRESAHA.110.232306 21311045 PMC3785241

[83] Pei H Wang W Zhao D Su H Su G Zhao Z. G protein-coupled estrogen receptor 1 inhibits angiotensin II-induced cardiomyocyte hypertrophy via the regulation of PI3K-akt-mTOR signalling and autophagy. Int J Biol Sci. (2019) 15(1):81–92. 10.7150/ijbs.28304 30662349 PMC6329915

[84] Zhan H Huang F Niu Q Jiao M Han X Zhang K Downregulation of miR-128 ameliorates ang II-induced cardiac remodeling via SIRT1/PIK3R1 multiple targets. Oxid Med Cell Longev. (2021) 2021:8889195. 10.1155/2021/8889195 34646427 PMC8505057

[85] Medina D Arnold AC. Angiotensin-(1-7): translational avenues in cardiovascular control. Am J Hypertens. (2019) 32(12):1133–42. 10.1093/ajh/hpz146 31602467 PMC6856625

[86] Flores-Muñoz M Godinho BMDC Almalik A Nicklin SA. Adenoviral delivery of angiotensin-(1-7) or angiotensin-(1-9) inhibits cardiomyocyte hypertrophy via the Mas or angiotensin type 2 receptor. PLoS One. (2012) 7(9):1–6. 10.1371/journal.pone.0045564 PMC3447802 23029101

[87] Senbonmatsu T Saito T Landon EJ Watanabe O Price E Roberts RL A novel angiotensin II type 2 receptor signaling pathway: possible role in cardiac hypertrophy. EMBO J. (2003) 22(24):6471–82. 10.1093/emboj/cdg637 14657020 PMC291832

[88] Sasaki Y Ikeda Y Iwabayashi M Akasaki Y Ohishi M. The impact of autophagy on cardiovascular senescence and diseases. Int Heart J. (2017) 58(5):666–73. 10.1536/ihj.17-246 28966332

[89] Sotomayor-Flores C Rivera-Mejías P Vásquez-Trincado C López-Crisosto C Morales PE Pennanen C Angiotensin-(1–9) prevents cardiomyocyte hypertrophy by controlling mitochondrial dynamics via miR-129-3p/PKIA pathway. Cell Death Differ. (2020) 27(9):2586–604. 10.1038/s41418-020-0522-3 32152556 PMC7429871

[90] Zhong T Wang Z Niloy SI Shen Y O’Rourke ST Sun C. Role of PI3-kinase in angiotensin II-induced cardiac hypertrophy: class I versus class III. Front Pharmacol. (2021) 12:1–11. 10.3389/fphar.2021.608523 PMC7921739 33664668

[91] Zhang Y Xu X Ceylan-Isik AF Dong M Pei Z Li Y Ablation of Akt2 protects against lipopolysaccharide-induced cardiac dysfunction: role of akt ubiquitination E3 ligase TRAF6. J Mol Cell Cardiol. (2014) 74:76–87. 10.1016/j.yjmcc.2014.04.020 24805195 PMC4115010

[92] DeBosch B Sambandam N Weinheimer C Courtois M Muslin AJ. Akt2 regulates cardiac metabolism and cardiomyocyte survival. J Biol Chem. (2006) 281(43):32841–51. 10.1074/jbc.M513087200 16950770 PMC2724003

[93] DeBosch B Treskov I Lupu TS Weinheimer C Kovacs A Courtois M Akt1 is required for physiological cardiac growth. Circulation. (2006) 113(17):2097–104. 10.1161/CIRCULATIONAHA.105.595231 16636172

[94] Zeng S-Y Yan Q-J Yang L Mei Q-H Lu H-Q. Inhibition of the ROS-EGFR pathway mediates the protective action of Nox1/4 inhibitor GKT137831 against hypertensive cardiac hypertrophy via suppressing cardiac inflammation and activation of akt and ERK1/2. Mediators Inflamm. (2020) 2020:1078365. 10.1155/2020/1078365 32831633 PMC7424508

[95] Gao S Park BM Cha SH Park WH Park BH Kim SH. Angiotensin AT2 receptor agonist stimulates high stretch induced-ANP secretion via PI3K/NO/sGC/PKG/pathway. Peptides. (2013) 47:36–44. 10.1016/j.peptides.2013.06.008 23791669

[96] Ma H Mao C Hu Y Wang L Guo X Li L Angiotensin-(1–9) attenuates adriamycin-induced cardiomyopathy in rats via the angiotensin type 2 receptor. Mol Cell Biochem. (2023) 479(1):73–83. 10.1007/s11010-023-04718-y 36995547

[97] Norambuena-Soto I Ocaranza MP Cancino-Arenas N Sanhueza–Olivares F Villar-Fincheira P Leiva–Navarrete S Angiotensin-(1-9) prevents vascular remodeling by decreasing vascular smooth muscle cell dedifferentiation through a FoxO1-dependent mechanism. Biochem Pharmacol. (2020) 180:114190. 10.1016/j.bcp.2020.114190 32768401

[98] Aita VM Liang XH Murty VVVS Pincus DL Yu W Cayanis E Cloning and genomic organization of beclin 1, a candidate tumor suppressor gene on chromosome 17q21. Genomics. (1999) 59(1):59–65. 10.1006/geno.1999.5851 10395800

[99] Qu X Yu J Bhagat G Furuya N Hibshoosh H Troxel A Promotion of tumorigenesis by heterozygous disruption of the beclin 1 autophagy gene. J Clin Invest. (2003) 112(12):1809–20. 10.1172/JCI20039 14638851 PMC297002

[100] Yue Z Jin S Yang C Levine AJ Heintz N. Beclin 1, an autophagy gene essential for early embryonic development, is a haploinsufficient tumor suppressor. Proc Natl Acad Sci U S A. (2003) 100(25):15077–82. 10.1073/pnas.2436255100 14657337 PMC299911

[101] Liang XH Jackson S Seaman M Brown K Kempkes B Hibshoosh H Induction of autophagy and inhibition of tumorigenesis by beclin 1. Nature. (1999) 402(6762):672–6. 10.1038/45257 10604474

[102] Levine B Liu R Dong X Zhong Q. Beclin orthologs: integrative hubs of cell signaling, membrane trafficking, and physiology. Trends Cell Biol. (2015) 25(9):533–44. 10.1016/j.tcb.2015.05.004 26071895 PMC4554927

[103] Pattingre S Tassa A Qu X Garuti R Xiao HL Mizushima N Bcl-2 antiapoptotic proteins inhibit beclin 1-dependent autophagy. Cell. (2005) 122(6):927–39. 10.1016/j.cell.2005.07.002 16179260

[104] Levine B Sinha S Kroemer G. Bcl-2 family members: dual regulators of apoptosis and autophagy. Autophagy. (2008) 4(5):600–6. 10.4161/auto.6260 18497563 PMC2749577

[105] Russell RC Tian Y Yuan H Park HW Chang YY Kim J ULK1 induces autophagy by phosphorylating beclin-1 and activating VPS34 lipid kinase. Nat Cell Biol. (2013) 15(7):741–50. 10.1038/ncb2757 23685627 PMC3885611

[106] Park JM Seo M Jung CH Grunwald D Stone M Otto NM ULK1 phosphorylates Ser30 of BECN1 in association with ATG14 to stimulate autophagy induction. Autophagy. (2018) 14(4):584–97. 10.1080/15548627.2017.1422851 29313410 PMC5959323

[107] Li X Wu XQ Deng R Li DD Tang J Chen WD CaMKII-mediated beclin 1 phosphorylation regulates autophagy that promotes degradation of id and neuroblastoma cell differentiation. Nat Commun. (2017) 8(1):1159. 10.1038/s41467-017-01272-2 29079782 PMC5660092

[108] Zhang D Wang W Sun X Xu D Wang C Zhang Q AMPK regulates autophagy by phosphorylating BECN1 at threonine 388. Autophagy. (2016) 12(9):1447–59. 10.1080/15548627.2016.1185576 27304906 PMC5082788

[109] Kim J Kim YC Fang C Russell RC Kim JH Fan W Differential regulation of distinct Vps34 complexes by AMPK in nutrient stress and autophagy. Cell. (2013) 152(1–2):290–303. 10.1016/j.cell.2012.12.016 23332761 PMC3587159

[110] Cheng Z Zhu Q Dee R Opheim Z Mack CP Cyr DM Focal adhesion kinase-mediated phosphorylation of Beclin1 protein suppresses cardiomyocyte autophagy and initiates hypertrophic growth. J Biol Chem. (2017) 292(6):2065–79. 10.1074/jbc.M116.758268 27994061 PMC5313082

[111] Decuypere J-P Parys JB Bultynck G. Regulation of the autophagic bcl-2/beclin 1 interaction. Cells. (2012) 1(3):284–312. 10.3390/cells1030284 24710477 PMC3901098

[112] Maiuri MC Le Toumelin G Criollo A Rain JC Gautier F Juin P Functional and physical interaction between bcl-XL and a BH3-like domain in beclin-1. EMBO J. (2007) 26(10):2527–39. 10.1038/sj.emboj.7601689 17446862 PMC1868901

[113] Ciechomska IA Goemans GC Skepper JN Tolkovsky AM. Bcl-2 complexed with beclin-1 maintains full anti-apoptotic function. Oncogene. (2009) 28(21):2128–41. 10.1038/onc.2009.60 19347031

[114] Chang NC Nguyen M Germain M Shore GC. Antagonism of beclin 1-dependent autophagy by BCL-2 at the endoplasmic reticulum requires NAF-1. EMBO J. (2010) 29(3):606–18. 10.1038/emboj.2009.369 20010695 PMC2830692

[115] Nishimura T Tooze SA. Emerging roles of ATG proteins and membrane lipids in autophagosome formation. Cell Discov. (2020) 6(1):32. 10.1038/s41421-020-0161-3 32509328 PMC7248066

[116] Li X He L Che KH Funderburk SF Pan L Pan N Imperfect interface of Beclin1 coiled-coil domain regulates homodimer and heterodimer formation with Atg14l and UVRAG. Nat Commun. (2012) 3:611–62. 10.1038/ncomms1613 22314358 PMC3293417

[117] Zeng C Zhang Z Luo W Wang L Zhou H Nie C. JNK initiates beclin-1 dependent autophagic cell death against akt activation. Exp Cell Res. (2022) 414(2):113105. 10.1016/j.yexcr.2022.113105 35306025

[118] Song X Lee DH Dilly AK Lee YS Choudry HA Kwon YT Crosstalk between apoptosis and autophagy is regulated by the arginylated BiP/beclin-1/p62 complex. Mol Cancer Res. (2018) 16(7):1077–91. 10.1158/1541-7786.MCR-17-0685 29669822 PMC6030503

