# Supplementary figures and images for: Autophagy fine-tuning by angiotensin-(1-9) in cultured rat cardiomyocytes
Source: Front Cardiovasc Med. 2025 Mar 12;12:1408325. doi: 10.3389/fcvm.2025.1408325 (PMC11937029; doi:10.3389/fcvm.2025.1408325)

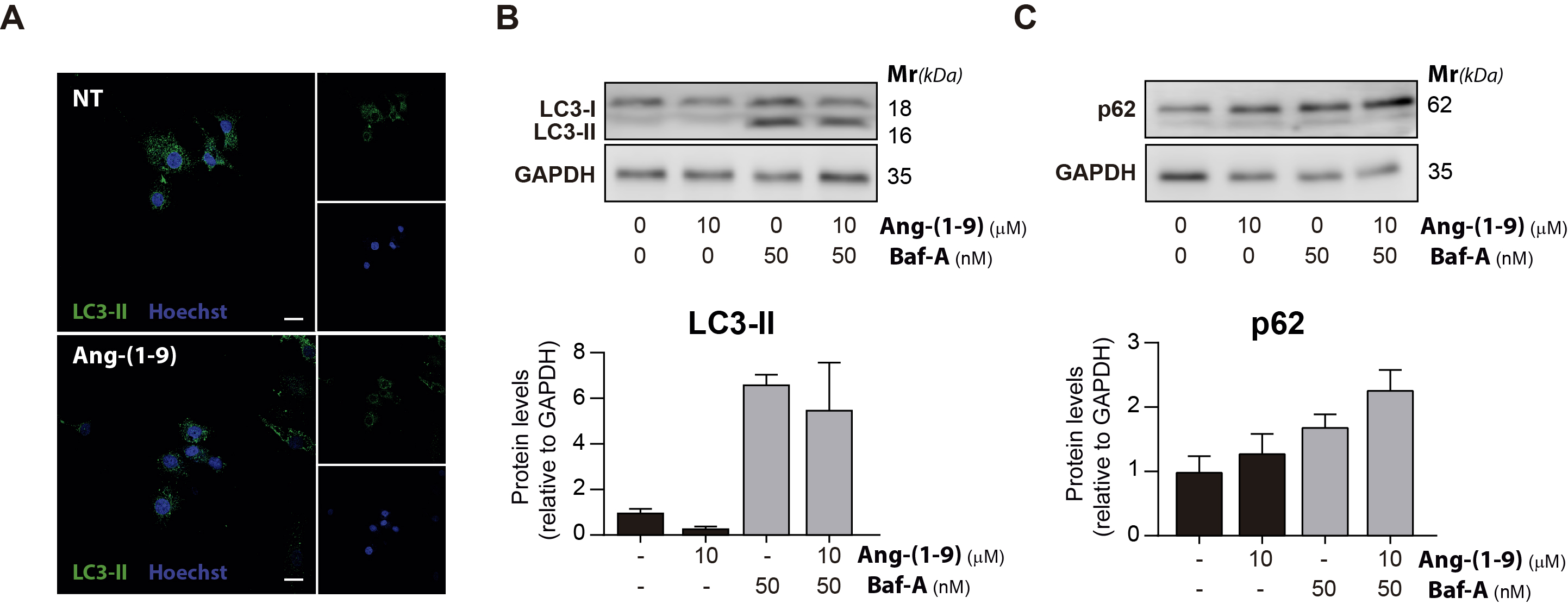

Supplement: Supplementary Figure S1 [file Image1.jpg]

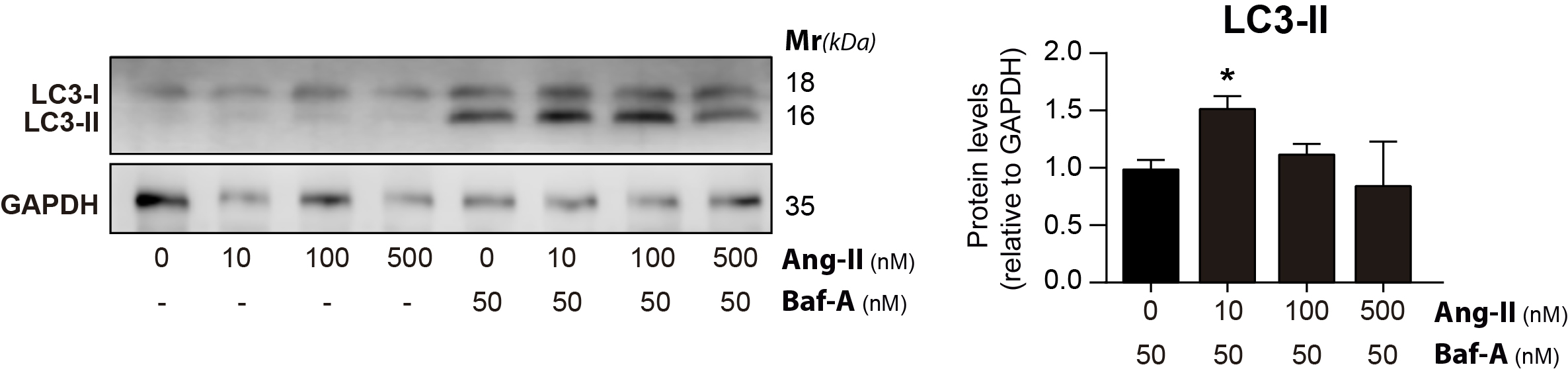

Supplement: Supplementary Figure S2 [file Image2.jpeg]

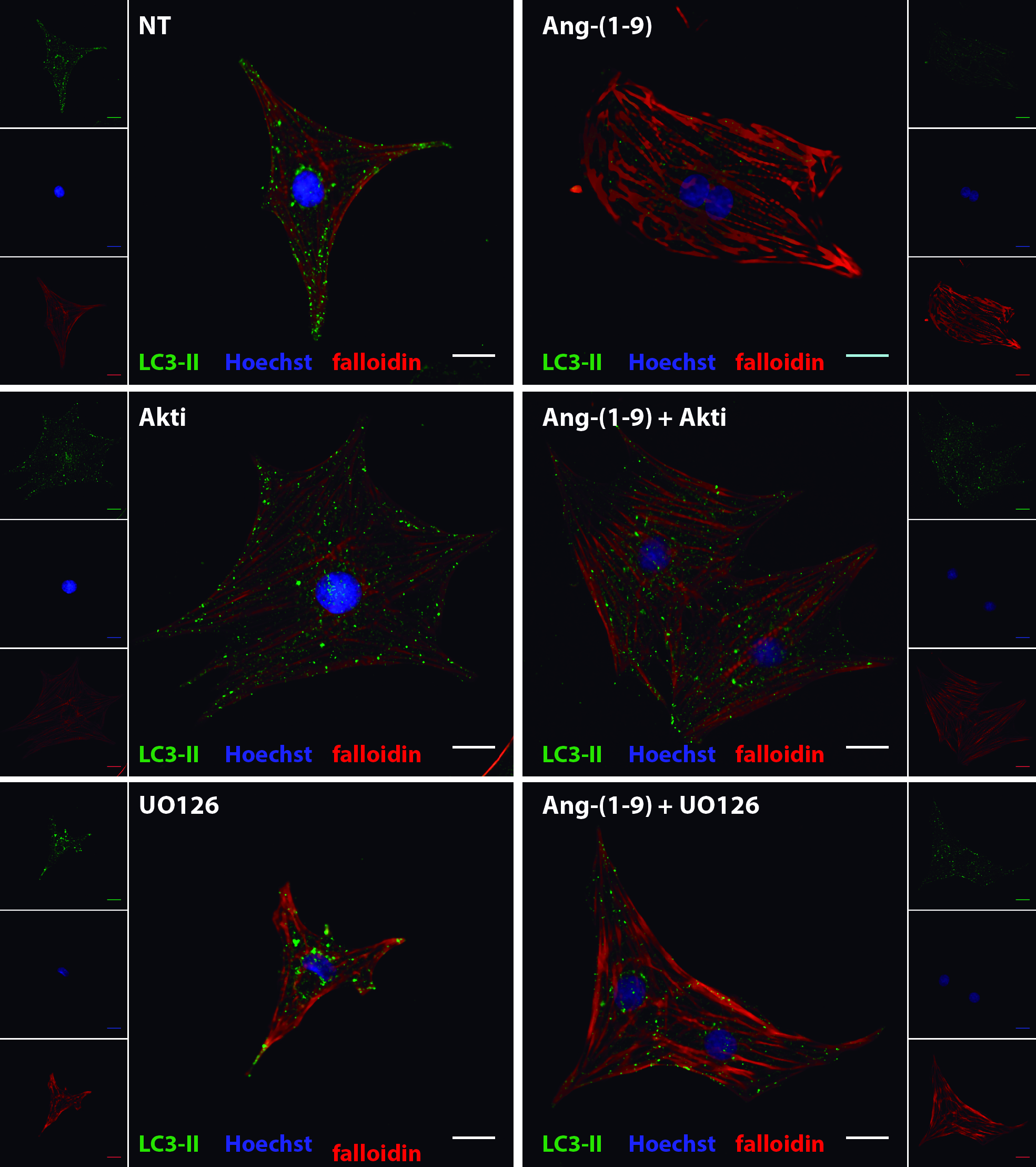

Supplement: Supplementary Figure S3 [file Image3.jpeg]
